# Herpes simplex virus type 2 seropositivity and relationship status among U.S. adults age 20 to 49: a population-based analysis

**DOI:** 10.1186/1471-2334-10-359

**Published:** 2010-12-22

**Authors:** Greta R Bauer, Nooshin Khobzi, Todd A Coleman

**Affiliations:** 1 Epidemiology & Biostatistics, Schulich School of Medicine & Dentistry, The University of Western Ontario, London, Ontario, Canada

## Abstract

**Background:**

U.S. population studies show herpes simplex virus type 2 (HSV-2) seroprevalence levelling by approximately age 30, suggesting few new infections after that age. It is unclear whether this pattern is driven by greater percentages in stable relationships, and to what extent adults who initiate new relationships may be at risk of incident HSV-2 infection.

**Methods:**

Survey and laboratory data from the 1999-2008 waves of the U.S. National Health and Nutrition Examination Survey (NHANES) were combined for 12,862 adults age 20-49. Weighted population estimates of self-reported genital herpes, HSV-2 seroprevalence, and past-year sexual history were calculated, stratified by age, sex, race, and relationship status. Multivariable logistic regression was used to assess whether relationship status provided additional information in predicting HSV-2 over age, race and sex, and whether any such associations could be accounted for through differences in lifetime number of sex partners.

**Results:**

Those who were unpartnered had higher HSV-2 prevalence than those who were married/cohabitating. Among unpartnered 45-49 year olds, seroprevalence was 55.3% in women and 25.7% in men. Those who were married/cohabitating were more likely to have had a past-year sex partner, and less likely to have had two or more partners. The effect of age in increasing the odds of HSV-2 was modified by race, with higher HSV-2 prevalence among Black Americans established by age 20-24 years, and the effect of race decreasing from age 30 to 49. Relationship status remained an independent predictor of HSV-2 when controlling for age, race, and sex among those age 30 to 49; married/cohabitating status was protective for HSV-2 in this group (OR = 0.69)

**Conclusions:**

Whereas sexually transmitted infections are often perceived as issues for young adults and specific high-risk groups, the chronic nature of HSV-2 results in accumulation of prevalence with age, especially among those not in married/cohabitating relationships. Increased odds of HSV-2 with age did not correspond with increases in self-reported genital herpes, which remained low. Adults who initiate new relationships should be aware of HSV-2 in order to better recognize its symptoms and prevent transmission.

## Background

While most research on sexually transmitted infections (STIs) has focused on adolescents, young adults, and established high risk groups, adults outside of these groups are not free of risk for STIs. The association between age and STI prevalence varies not only by population, but by type of infection. As prevalence is a function of both incidence rate and duration of infection, prevalence of chronic viral infections such as genital herpes is cumulative and increases with age,[1-6] thus possessing special relevance for those beyond the "young adult" years.

Genital herpes results from infection with herpes simplex virus type 2 (HSV-2), or less commonly type 1 (HSV-1). Since HSV-1 is responsible for most oral outbreaks or "cold sores" and represents a minority of genital infections, HSV-2 is used in seroprevalence studies as a marker of burden of infection with genital herpes. In the U.S., HSV-2 seroprevalence increased 30% from 1976-80 to 1988-94,[1] and decreased by 19% from 1988-94 to 1999-2004 in those aged 14 to 49 years, with the strongest decreases among those aged 14 to 19[7].

A number of population-based prevalence studies of age-related trends in HSV-2 have been conducted, with age-related patterns varying across populations[8]. In the U.S., HSV-2 prevalence increased until approximately age 30, and then stabilized[1]. In Australia, prevalence similarly peaked at midlife and then plateaued[5]. In Ontario, Canada, HSV-2 prevalence did not stabilize but rather continued to increase through the oldest group studied - 40 to 44 years - a pattern suggesting additional new infections among middle-aged adults,[2] and in Puerto Rico prevalence stabilized after age 40[6]. Countries such as Costa Rica and Switzerland have observed the highest prevalences among the most elderly,[3,4] though findings of highest prevalence in elderly men in Switzerland were believed to represent a World War II cohort effect[4]. Age trends observed in cross-sectional studies may be due to cohort effects, different rates of infection in different generations. However, it is likely that an increase in prevalence with age also represents new infections occurring at older ages.

Slower rates of acquisition of new infections in older adults vs. young adults as a whole are at least in part due to the increase in stable long-term relationships with age. By age 30, approximately 60% of U.S. adults are married, and this proportion remains relatively stable in groups up to age 65 (Stevenson, 2007). However, stable population proportions for marriage do not necessarily correspond to stable marriages among individuals. Changes in relationship and family structure have resulted in increasing proportions of adults finding themselves outside of long-term monogamous relationships, at least for periods of time. The divorce rate in the United States rose sharply until 1981 and has recently levelled off[9]. The marriage rate also decreased over the past 25 years, and is now at its lowest point in recorded history, representing new ways to envision relationships[9]. Cohabitation between unmarried partners has increased ten-fold between 1960 and 2000, with an 88% increase between 1990 and 2007[10]. Over the life course, relationship changes due to divorce, separation or death lead to new patterns of casual dating, short-term serial monogamy, new long-term relationships, or remarriage.

While overall HSV-2 prevalence in adults is high, we hypothesize it may be even higher in the pool of people from which new partners are typically drawn: those who are not married or currently cohabitating with a partner. Thus, for middle-aged and older adults, the probability of a new partner being infected with a chronic infection such as HSV-2 may be higher than for other groups, despite a lower risk of curable STIs, which are of shorter duration and thus at lower prevalence. Models for transmission of STIs frequently incorporate individual behavioural factors, such as rate of partner change and concurrent partnerships. However, prevalence of infection in the prospective partner pool alone can result in widely different outcomes for STI incidence. For example, it has been demonstrated that men who have sex with other men (MSM) would have to be much safer than heterosexuals in order to stem the HIV epidemic and that heterosexuals would conversely have to behave in much riskier ways than MSM to produce a sustained epidemic[11]. Likewise, African-American youth have been demonstrated to be at high risk for STIs, even when engaging in "low-risk" sexual and drug behaviours[12]. In both of these cases, similar behaviours are believed to result in very different risks of infection primarily due to the different probabilities of coming into contact with an infected partner. In general, HSV-2 seropositivity varies by age, race and sex in the U.S. population[1,7,8,13,14]. Currently, the prevalence of HSV-2 among unpartnered adults, the group from which new sex partners would most likely be chosen, is unknown; exploring this is one aim of the current analysis. The present study seeks to further explore whether relationship status provides any valuable additional information beyond these three factors in predicting HSV-2.

## Methods

### Data

Data were obtained from the National Health and Nutrition Examination Survey (NHANES). NHANES is a continuous sample survey that uses a complex, multistage, stratified geographic area probability sampling design for collecting nationally representative data from the non-institutionalized U.S. population[15]. Cross-sectional data were collected in two-year waves, and data from five waves between 1999 and 2008 were combined for this analysis in order to produce large sample sizes for analysis.

Demographic information, including age, sex, race/ethnicity, and relationship status, was obtained through in-home interviews in English or Spanish. More sensitive survey items such as self-reported genital herpes and sexual history were assessed at a local Mobile Examination Center (MEC) using audio computer-assisted self-interview, wherein participants listened to questions on headphones and input their responses directly into a computer in a private room, without interviewer observation. This method has been shown to result in higher reporting of sensitive behaviours[16,17].

Laboratory specimens for HSV-2 testing were obtained at the MEC. Sera from respondents aged 14-49 years were tested for antibody to HSV-2 infection and test results for those over age 18 were released. Sexual behaviour and infection questionnaire data were obtained for participants 18-59 years; however, only data from those 20 to 59 years of age were released. Our analyses were thus limited to MEC participants aged 20-49 years. Of 51,623 NHANES participants in the ten-year period from 1999 to 2008, 13,465 were between the age of 20 and 49, and of these, 12,862 (95.5%) participated in the MEC exam.

### Measures

Relationship status was assessed using the following categories: never married, married, living with a partner, divorced, and widowed. For this analysis, those who were never married, divorced or widowed were grouped together as "unmarried" to represent the population most likely to be available for or initiating new sexual relationships. Those who were married or cohabitating with a partner were classified as "married/cohabitating" to represent those unlikely to be initiating new sexual relationships. Race/ethnicity was measured as Mexican American, other Hispanic, non-Hispanic white, non-Hispanic Black, and other (including multiracial). For our analyses, Mexican Americans and other Hispanics were jointly categorized as Hispanic. In the course of our analysis, and consistent with published literature, we found differences in HSV-2 seropositivity and in the unadjusted rate of increase of prevalence with age between Black respondents and either Hispanic or White respondents. As no differences were found between White, Hispanic and "Other" groups, these were grouped together in analysis for simplicity of presentation and to increase power to examine interactions in regression models.

Measures of sexual activity included having any sex in the past year, and having two or more sex partners in the past year. Sex was defined as vaginal, anal or oral sex with a male or female partner. Lifetime number of sex partners was calculated by summing the total number of male partners and female partners.

Two measures of genital herpes were used for our analyses. First, self-reported genital herpes was assessed with the following questionnaire item: "Has a doctor or other health care professional ever told you that you had genital herpes?" Second, laboratory assessment of HSV-2 was conducted in serum samples using a solid-phase enzymatic immunodot assay to detect antibodies reactive to gG-2, a purified glycoprotein specific to HSV-2; positive immunodot assays were confirmed using a gG-2 monoclonal antibody inhibition assay[18]. The same assays were used over the 1999-2008 survey cycles, and in the previous NHANES III[18].

### Analysis

All statistical analyses were conducted with SAS version 9.2 [19] using SURVEYFREQ and SURVEYLOGISTIC procedures designed to deal with complex survey data. Prevalence estimates were derived using 10-year weights specific to the MEC sample, to represent the U.S. civilian, non-institutionalized population of adults aged 20-49 years, and to account for oversampling and nonresponse to the interview and medical examinations. Primary sampling unit, stratum and individual weights were used to produce weighted point estimates, and variances were calculated using Taylor series linearization.

Frequencies for being unmarried were calculated by age in five-year intervals. All analyses were stratified according to age, sex and race. Age-, sex-, race- and relationship status-specific prevalences for HSV-2 seropositivity and self-reported genital herpes were calculated, along with corresponding 95% confidence intervals. A similar domain analysis was conducted for sexual activity variables.

Multivariable unconditional logistic regression was used to elucidate the strength and combination of effects on HSV-2 seropositivity as follows. A model was fit incorporating age, sex, race, and any interactions between these variables that were statistically significant (p < 0.05 for Wald chi-square test). Where any interaction was significant, lower-order terms were retained in the model regardless of level of statistical significance. Relationship status was then added to the model, to test whether it contributed anything to predicting HSV-2 seropositivity beyond age, race and sex. Interactions between relationship status and age, race and sex were tested, and interaction terms retained where statistically significant or where marginally significant if effect size was large. Finally, to test whether any association between relationship status and HSV-2 seropositivity may be explained by differences in lifetime number of sex partners, this variable was entered into the model. Lifetime sex partner number was coded into five ordered categories, to allow for a non-linear association between sex partner number and log odds of HSV-2.

Given that previous analysis of U.S. data had shown HSV-2 seroprevalence to level off at approximately age 30,[1] and since our primary variable of interest, relationship status, exhibited an interaction with age, we conducted a sensitivity analysis to see whether regression results were heavily influenced by the youngest adults in our analysis, those aged 20-29 years. A second logistic regression model was built according to the process above, but limited to participants aged 30-49 years. As this model differed from that containing the full sample, separate regression models were presented for those age 20-29 and those age 30-49.

## Results

Our analyses included 5,943 males and 6,919 females. The percentage of Americans who were unpartnered decreased markedly with increasing age. At age 20-24, 63.3% of women and 73.9% of men remained unmarried and were not cohabitating with a partner. This proportion decreased sharply through age 29, and by age 45-49, only 29.8% of women and 24.8% of men were unpartnered. Among unpartnered women, the overall prevalence of self-reported genital herpes was low (5.8%; 95% CI: 4.5, 7.2) and did not differ significantly from married/cohabitating women (5.7%; 95% CI: 4.8, 6.6). However, overall seropositivity for HSV-2 was 32.3% (95% CI: 29.8, 34.8) for unpartnered women, significantly higher than the 22.3% (95% CI: 20.7, 23.9) estimated for married/cohabitating women. Among men, HSV-2 seroprevalence overall did not differ by relationship status. Overall HSV-2 seroprevalence was 13.6% (95% CI: 11.8, 15.4) among unpartnered men, and 13.0% (95% CI: 11.3, 14.7) among married/cohabitating men. Similarly to women, the prevalence of self-reported genital herpes was much lower than HSV-2 seroprevalence, with 2.3% (95% CI: 1.6, 3.0) of unpartnered and 2.3% (95% CI: 1.6, 3.0) of married/cohabitating men reporting genital herpes.

**Figure 1:**
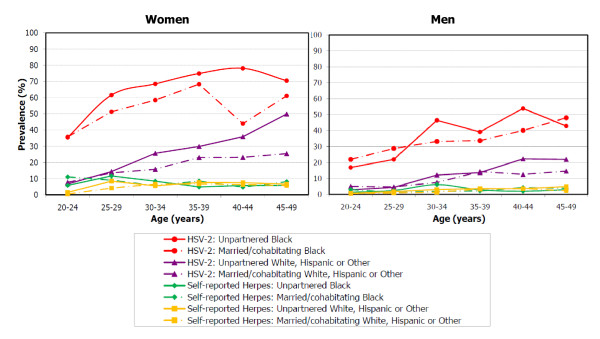
**Prevalence of herpes simplex virus type 2 seropositivity and self-reported genital herpes by age, race, and relationship status: U.S. women and men, 1999-2008**.

Age-, race-, sex-, and relationship status-specific prevalences for HSV-2 and self-reported genital herpes are illustrated in Figure 1. Specific prevalences and corresponding 95% confidence intervals are reported in Table 1 for HSV-2 and Table 2 for self-reported genital herpes. Self-reported prevalences of genital herpes remained low across all ages, regardless of race, sex, or relationship status. At age 45-49, estimates of self-reported genital herpes ranged from 2.2% among married/cohabitating white/Hispanic/other men to 8.2% among married Black women. However, the seroprevalence of HSV-2 increased with age, particularly among unpartnered women, resulting in increasing discrepancies between self-reported genital herpes and HSV-2 seropositivity with age. At age 45-49 years, estimated HSV-2 seroprevalences for women were: 61.1% among married Blacks, 70.5% among unpartnered Blacks, 25.5% among married white/Hispanic/others, 49.9% among unmarried white/Hispanic/others. The corresponding prevalences for men were: 48.0%, 42.9%, 14.6%, and 22.0%. While age- and relationship-related patterns for HSV-2 appeared similar across race groups, Black American men and women had substantially higher HSV-2 prevalence than those of white, Hispanic or other racial groups at age 20-24, resulting in higher prevalences across ages. Disparities in early HSV-2 seroprevalence were especially marked for Black women, with a prevalence of 35.1% (95% CI: 27.5, 42.6) at age 20-24, versus 7.1% (95% CI: 4.8, 9.4) for white, Hispanic or other women.

**Table 1:** Seroprevalences of herpes simplex virus type 2 among U.S. women and men, by age, relationship status and race

	Herpes simplex type 2 seropositivity, % (95% CI)					
	
	Black			White, Hispanic or Other		
	
	Married or cohabitating (n = 483)	Unpartnered (n = 687)	Total (n = 1170)	Married or cohabitating (n = 956)	Unpartnered (n = 524)	Total (n = 1480)
	
Women (n) Age, y						
**20-24 (167)**	35.8 (21.7, 49.8)	35.5 (27.8, 43.3)	35.1 (27.5, 42.6)	7.8 (4.3, 11.3)	6.8 (4.0, 9.6)	7.1 (4.8, 9.4)

**25-29 (243)**	51.3 (39.2, 63.5)	61.6 (52.6, 70.7)	58.0 (50.6, 65.5)	13.6 (10.0, 17.1)	14.4 (9.2, 19.7)	13.9 (11.0, 16.8)

**30-34 (294)**	58.5 (47.7, 69.2)	68.5 (59.0, 78.0)	63.8 (56.3. 71.3)	15.8 (12.5, 19.1)	25.7 (18.1, 33.4)	18.5 (15.2, 21.9)

**35-39 (322)**	68.3 (58.0, 78.6)	74.9 (64.6, 85.2)	71.3 (63.4, 79.3)	23.1 (19.1, 27.1)	29.9 (22.7, 37.2)	25.3 (21.6, 29.1)

**40-44 (360)**	44.0 (33.9, 54.2)	78.1 (70.3, 85.8)	62.9 (55.6, 70.1)	23.2 (18.9, 27.5)	35.9 (28.6, 43.2)	26.6 (22.7, 30.6)

**45-49 (384)**	61.1 (50.5, 71.6)	70.5 (61.8, 79.1)	66.5 (59.1, 73.9)	25.5 (21.0, 29.9)	49.9 (43.3, 56.6)	31.4 (27.8, 35.0)

**Men (n)** **Age, y**						

**20-24 (62)**	22.0 (5.9, 38.1)	16.9 (11.8, 21.9)	18.2 (13.1, 23.4)	5.0 (1.3, 8.7)	2.8 (1.2, 4.3)	3.4 (1.9, 4.9)

**25-29 (76)**	28.8 (19.4, 38.1)	22.0 (12.0, 32.1)	25.1 (18.4, 31.8)	4.7 (2.4, 7.1)	4.4 (2.1, 6.8)	4.8 (3.1, 6.5)

**30-34 (129)**	33.2 (24.6, 41.9)	46.5 (36.2, 56.8)	38.5 (32.5, 44.6)	7.4 (4.8, 10.1)	12.1 (6.6, 17.6)	8.9 (6.7, 11.1)

**35-39 (172)**	33.7 (24.1, 43.2)	39.2 (24.6, 53.9)	36.8 (28.8, 44.9)	14.3 (10.8, 17.9)	13.6 (7.9, 19.3)	14.1 (11.3, 16.9)

**40-44 (215)**	40.1 (32.1, 48.1)	53.9 (43.1, 64.7)	45.4 (39.1, 51.7)	12.5 (9.0, 16.1)	22.3 (14.7, 29.8)	15.2 (12.2, 18.3)

**45-49 (226)**	48.0 (38.9, 57.1)	42.9 (34.2, 51.6)	46.2 (39.6, 52.9)	14.6 (10.8, 18.4)	22.0 (14.0, 30.1)	16.6 (13.1, 20.1)

**Table 2:** Prevalences of self-reported genital herpes among U.S. women and men, by age, relationship status and race

	Self-reported genital herpes, % (95% CI)					
	
	Black			White, Hispanic or Other		
	
	Married or cohabitating (n = 51)	Unpartnered (n = 69)	Total (n = 120)	Married or cohabitating (n = 180)	Unpartnered (n = 95)	Total (n = 275)
**Women (n)** **Age, y**						

**20-24 (24)**	11.1 (2.3, 20.0)	5.9 (2.3, 9.4)	6.9 (3.3, 10.5)	0.5 (0.0, 1.0)	1.5 (0.0, 2.9)	1.1 (0.2, 2.0)

**25-29 (56)**	9.1 (1.1, 17.0)	11.7 (5.9, 17.5)	10.5 (6.1, 14.9)	4.2 (1.9, 6.5)	8.5 (3.6, 13.4)	5.7 (3.3, 8.0)

**30-34 (49)**	5.8 (0.7, 10.8)	8.5 (2.7, 14.2)	6.9 (3.4, 10.4)	6.7 (3.8, 9.5)	5.6 (1.1, 10.2)	6.6 (4.1, 9.0)

**35-39 (52)**	8.6 (4.5, 12.8)	4.9 (0.9, 9.0)	7.3 (4.2, 10.4)	6.8 (4.4, 9.1)	7.7 (3.1, 12.4)	6.8 (4.8, 8.8)

**40-44 (52)**	4.8 (0.1, 9.4)	4.8 (0.4, 9.1)	4.7 (1.4, 8.0)	6.4 (3.9, 9.0)	7.6 (3.2, 11.9)	6.9 (4.7, 9.1)

**45-49 (51)**	8.2 (2.6, 13.8)	5.9 (1.1, 10.8)	7.1 (3.1, 11.1)	5.8 (3.4, 8.2)	6.7 (2.3, 11.2)	5.9 (3.9, 7.9)

**Men (n)** **Age, y**						

**20-24 (6)**	3.0 (0.0, 8.7)	1.2 (0.0, 2.8)	2.2 (0.0, 4.3)	0.5 (0.0, 1.4)	0.5 (0.0, 1.2)	0.5 (0.0, 1.1)

**25-29 (10)**	1.2 (0.0, 3.7)	2.4 (0.0, 5.9)	1.9 (0.0, 47.1)	0.9 (0.0, 2.1)	1.7 (0.0, 3.7)	1.2 (0.1, 2.3)

**30-34 (19)**	1.9 (0.0, 4.6)	6.1 (0.9, 11.3)	3.7 (1.0, 6.4)	1.3 (0.1, 2.5)	3.0 (0.0, 5.9)	1.7 (0.6, 2.8)

**35-39 (23)**	2.1 (0.0, 5.2)	2.8 (0.0, 6.9)	2.7 (0.2, 5.2)	3.3 (1.4, 5.3)	3.6 (0.3, 6.9)	3.8 (2.1, 5.5)

**40-44 (28)**	4.3 (0.8, 7.7)	1.9 (0.0, 4.7)	3.2 (0.9, 5.4)	3.6 (1.6, 5.6)	3.5 (0.1, 7.0)	3.4 (1.8, 5.1)

**45-49 (25)**	3.8 (0.2, 7.3)	2.9 (0.0. 6.4)	3.3 (0.8, 5.9)	2.2 (0.6, 3.7)	4.9 (0.8, 9.0)	3.0 (1.4, 4.6)

Weighted frequencies and corresponding 95% confidence intervals for sexual history variables are presented in Table 3. Across all groups, married/cohabitating individuals were more likely to have had sex in the past year than those who were unpartnered, and unpartnered individuals were more likely to have had two or more sex partners.

**Table 3:** Prevalences of past-year sexual activity and partner number among U.S. women and men, by age, relationship status and race

	**Any sex**^**a **^**partner in the past year, % (95% CI)**				**2 or more sex**^**a **^**partners in the past year, % (95% CI)**			
	
	Black		White, Hispanic or Other		Black		White, Hispanic or Other	
	
	Married or cohabitating (n = 968)	Unpartnered (n = 1064)	Married or cohabitating (n = 5409)	Unpartnered (n = 2127)	Married or cohabitating (n = 173)	Unpartnered (n = 536)	Married or cohabitating (n = 418)	Unpartnered (n = 933)
**Women** **Age, y**								

**20-24**	100.0 (100.0, 100.0)	89.4 (84.6, 94.2)	99.2 (98.4, 100.0)	72.6 (66.7, 78.6)	15.0 (4.7, 25.3)	34.0 (25.9, 42.1)	10.3 (6.7, 14.0)	29.1 (24.0, 34.1)

**25-29**	96.0 (91.0, 100.0)	83.2 (76.3, 90.1)	99.6 (99.1, 100.0)	83.4 (78.1, 88.8)	17.1 (6.0, 28.2)	36.4 (27.0, 45.8)	8.6 (5.7, 11.6)	40.4 (34.2, 46.7)

**30-34**	97.8 (94.7, 100.0)	87.4 (82.4, 92.3)	98.4 (97.2, 99.6)	74.5 (65.8, 83.2)	11.1 (4.9, 17.2)	36.6 (28.7, 44.4)	7.1 (4.3, 9.8)	30.4 (21.8, 38.9)

**35-39**	98.8 (97.0, 100.0)	79.3 (70.9, 87.8)	98.1 (96.4, 99.8)	78.8 (72.8, 84.7)	9.9 (3.5, 16.4)	29.1 (20.4, 37.9)	4.1 (2.2, 5.9)	26.2 (19.1, 33.3)

**40-44**	97.0 (92.7, 100.0)	80.3 (73.7, 86.8)	97.2 (95.1, 99.2)	62.9 (55.0, 70.8)	6.8 (0.8, 12.8)	21.1 (13.7, 28.4)	3.6 (1.9, 5.4)	20.9 (14.9, 26.9)

**45-49**	93.8 (89.5, 98.1)	62.6 (51.6, 73.6)	93.7 (91.4, 96.0)	54.7 (45.5, 63.8)	11.8 (4.4, 19.1)	18.8 (10.8, 26.7)	2.8 (1.4, 4.2)	11.6 (6.3, 17.0)

**Men** **Age, y**								

**20-24**	97.0 (91.2, 100.0)	83.1 (78.0, 88.2)	99.3 (98.4, 100.0)	76.9 (72.3, 81.5)	23.9 (9.2, 38.6)	59.5 (53.3, 65.7)	16.4 (9.8, 22.9)	44.5 (39.8, 49.3)

**25-29**	94.1 (88.2, 100.0)	90.9 (84.9, 96.9)	97.9 (96.8, 99.1)	78.9 (74.9, 82.9)	26.7 (14.9, 38.6)	61.0 (51.3, 70.6)	12.8 (9.7, 15.9)	41.0 (34.3, 47.6)

**30-34**	94.9 (90.4, 99.4)	83.6 (72.8, 94.4)	98.1 96.5, 99.7)	77.9 (70.6, 85.2)	20.1 (10.3, 29.8)	57.1 (43.8, 70.5)	7.8 (4.9, 10.7)	45.7 (35.6, 55.7)

**35-39**	96.7 (92.7, 100.0)	78.7 (68.4, 88.9)	95.9 (94.4, 97.3)	76.3 (67.2, 85.5)	25.8 (17.3, 34.3)	46.2 (34.5, 57.8)	6.5 (4.6, 8.4)	32.2 (23.2, 41.1)

**40-44**	96.8 (93.6, 100.0)	77.0 (66.5, 87.5)	96.1 (94.2, 98.1)	70.4 (61.9, 78.9)	18.7 (10.4, 27.0)	47.3 (35.4, 59.2)	6.2 (4.0, 8.3)	33.2 (25.0, 41.5)

**45-49**	97.2 (94.0, 100.0)	82.4 (73.2, 91.5)	95.5 (93.4, 97.6)	62.4 (53.0, 71.8)	26.1 (17.5, 34.6)	56.1 (44.0, 68.3)	4.0 (2.5, 5.4)	24.2 (17.2, 31.2)

^a^Sex is defined as oral, vaginal or anal sex with a male or female partner.

Results from multiple logistic regression models predicting HSV-2 seropositivity among that age 20-29 and 30-49 are presented in Table 4. For 20-29 year olds, being married or cohabitating was not associated with HSV-2 seropositivity, controlling for age, race, sex, and lifetime sex partner number. In contrast, among 30-49 year olds, those who were married or cohabitating had a statistically significant decreased odds of HSV-2 (OR = 0.69) Within the 30 to 49 year old age stratum, the association between relationship status and HSV-2 was not explained by the total number of lifetime sex partners. In both models, increasing age, Black race, and female sex were associated with increased odds of HSV-2 seroprevalence, controlling for lifetime sex partner number and relationship status, where statistically significant. However, among those age 30 to 49, age was associated with increased seroprevalence of HSV-2 in White, Hispanic and other-raced people, but not in Blacks, as the relative impact of Black race decreased with increasing age, corresponding to odds ratios of 6.05 at age 30, 4.20 at age 40, and 3.02 at age 49.

**Table 4:** Logistic regression models, U.S. adults aged 20 to 29 and aged 30 to 49 years

	Parameter Estimate (β)	Standard Error	p	OR	95% CI
**Ages 20-29**					

**Intercept**	-7.2449	0.7268	< 0.0001	-	-

**Age, y**	0.1327	0.0262	< 0.0001	1.14	(1.09, 1.20)

**Sex ^a^**	1.1769	0.1483	< 0.0001	3.24	(2.43, 4.34)

**Race ^b^**	1.7669	0.1410	< 0.0001	5.85	(4.44, 7.72)

**Lifetime partners: 1 vs. 0**	-0.7554	0.4934	0.1258	0.47	(0.18, 1.24)

**Lifetime partners: 2 vs. 0**	-0.2113	0.5046	0.6754	0.81	(0.30, 2.18)

**Lifetime partners: 3-5 vs. 0**	0.6451	0.3854	0.0942	1.91	(0.90, 4.06)

**Lifetime partners: 6+ vs. 0**	1.1957	0.3642	0.0010	3.31	(1.62, 6.75)

**Ages 30-49**					

**Intercept**	-3.9644	0.4185	< 0.0001	-	-

**Age, y**	0.0481	0.00758	< 0.0001	^d^	(1.03, 1.07)

**Sex ^a^**	1.0727	0.0799	< 0.0001	2.92	(2.50, 3.42)

**Race ^b^**	2.8982	0.4780	< 0.0001	^d^	(7.11, 46.29)

**Relationship status ^c^**	-0.3680	0.0803	< 0.0001	0.69	(0.59, 0.81)

Age*Race interaction	-0.0366	0.0119	0.0021	^d^	(0.94, 0.99)

**Lifetime partners: 1 vs. 0**	-1.4657	0.2592	< 0.0001	0.23	(0.14, 0.38)

**Lifetime partners: 2 vs. 0**	-0.4669	0.2805	0.0960	0.63	(0.36, 1.09)

**Lifetime partners: 3-5 vs. 0**	0.1392	0.2068	0.5010	1.15	(0.77, 1.72)

**Lifetime partners: 6+ vs. 0**	0.6846	0.2188	0.0018	1.98	(1.29, 3.05)

a Coded 1 = female, 0 = male

b Coded 1 = Black, 0 = White, Hispanic or other

c Coded 1 = married/cohabitating, 0 = other

d An interaction between age and race resulted in varying ORs. For age, OR = 1.05 per year of age for white, Hispanic and others and OR = 1.01 per year of age for Blacks. For race, OR = 6.05 at age 30, OR = 4.20 at age 40, and OR = 3.02 at age 49.

## Discussion

Using interview and laboratory data from a large stratified probability sample of the U.S. population, this study demonstrates high HSV-2 seroprevalence among adults in general, and disparities in infection prevalences according to race and relationship status. Relationship status was found to be an independent predictor of HSV-2 seroprevalence among adults age 30 to 49, even when lifetime number of partners was taken into account, though relationship status no longer had a significant effect at ages 20 to 29 once partner number was included in the model. Our findings indicate that relationship status provides a moderate amount of additional information about the risk of having contracted HSV-2 among U.S. adults. Past the age of 30, the group from which sex partners are most often drawn - those not married or living with a partner - has higher odds of HSV-2 seropositivity than their married/cohabitating peers, controlling for demographic factors and partner number. As such, the impact of partnering status should be considered in future studies of HSV-2 seroprevalence.

Despite strengths related to the size and probability-based nature of our data set, these analyses have several data-related limitations. To achieve a large sample size, we pooled data collected over a ten-year period. Thus, our analysis assumes that results would be consistent over this time period. As our estimates combined married and cohabitating individuals, proportions with two or more sex partners in the past year are higher in our analysis than in U.S. estimates that included only married individuals, which estimate that 4.5% of married men and 3.8% of married women have had multiple past-year sex partners[20]. While those who were married or cohabitating were on average less likely to be infected with HSV-2, we were unable to separate out those who were monogamous from those who were overtly or covertly non-monogamous. The measure for multiple partnerships covers a one-year period; thus it represents a combination of concurrent partnerships within marriage or cohabitation, either due to open relationships or infidelity; concurrency before marriage or cohabitation; and serial partnerships with a partner change during the past year. We caution against the assumption of lower risk for a partner who is married or cohabitating, as it is likely that the non-monogamous subgroup does not share the same risk profile and HSV-2 prevalence of the monogamous subgroup. Estimated frequencies for having multiple past-year partners were higher for those who were unpartnered than for those who were married or cohabitating. The proportion of unpartnered women indicating multiple past-year partners decreased with age, similar to findings from the 2002 National Survey of Family Growth[21]. Among men, that decrease was limited to the white/Hispanic/other group.

HSV-2 seroprevalence and sexual behaviour data were collected only for those up to age 49 and 59, respectively. Fleming et al. showed consistent and stabilized HSV-2 seroprevalence among U.S. adults over age 30 using earlier NHANES III data that did not have this age restriction[1]. Given the heterogeneity within this group, inclusion of laboratory tests for HSV-2 among adults over age 49 would have allowed for expansion of the current analysis into older age ranges. While misconceptions and stereotypes persist, most single individuals desire to date and continue to be sexually active as they age[22]. In a U.S. survey of 3501 single individuals age 40 to 69, 31% were exclusively dating one person and 32% dating more than one person during the same time period[23]. With an aging baby boomer population, and the cultural shift brought on by the sexual revolution of the 1960s and -70s, conversations about middle-aged and older adult sexuality are becoming more common. Additionally, new treatments for erectile dysfunction - estimated to affect 20% of older males [24] - have prolonged sexual functioning. With this, the U.S. has seen a roughly 87% increase in prescriptions for erectile dysfunction medications from 1998 to 2001[25]. The cultural reform resulting from the popularity of the internet has also allowed older adults to express, experiment and challenge popular notions of asexuality and sexual disinterest[26]. Thus, given trends observed up to age 49, and the knowledge that adults over 50 remain sexually active, it is likely that genital herpes is a concern for this group as well.

Our analysis found overall HSV-2 rates to stabilize with age, consistent with results from recent analyses of 1999-2004 NHANES data,[7] and earlier examinations of NHANES II and NHANES III data[1]. Prevalences were higher among women than men in our analysis, and among Black Americans than other races, similar to findings from other studies[1,7,8,13,14]. However, the increase in HSV-2 with age was not significantly different for Blacks than for other racial groups across the 20-29 year age range, and in fact increased at a slower rate over ages 30-49. This indicates that disparities in HSV-2 infection between Black Americans and other racial groups in the U.S. are driven primarily by high rates of HSV-2 acquisition at younger ages. By age 20-24, seropositivity had reached 35.1% (95% CI: 27.5, 42.6) and 18.2% (95% CI: 13.1, 23.4) for Black women and men in our analysis, respectively.

Explanations for disparities in HSV-2 between Black and other Americans have been varied. While inequalities in access to STI treatment may account for disparities in bacterial STIs, they are less likely to account for differences in viral STIs such as HSV-2[27]. Individual behavioural and socioeconomic factors likely account for some of the disparity, though these have limited explanatory power, especially among low-risk Black Americans. The relative effect of non-Hispanic Black race/ethnicity has been shown to be strongest among a low-risk group,[12,13] an effect that was not reduced or eliminated by adjustment for socio-economic factors[13]. Even among those having only one lifetime sex partner, age-adjusted HSV-2 prevalence among Black men and women has been shown to be 4.4 times that of whites[1]. Differences in sexual network composition can produce different STI risks at the same behavioural risk levels, and such network composition differences for Black Americans include greater network density, higher rates of concurrent partnerships, and higher rates of mixing between core (i.e. high risk) groups and others[28]. These network patterns may be driven by social context, including racial segregation, low sex ratios of men to women, the effects of the crack cocaine epidemic of the 1980s, and high incarceration rates among Black U.S. men[28,29]. In our analysis, approximately twice as many Black men as women reported having two or more past-year sex partners, even among those who were married or cohabitating. It has been suggested that for Black Americans, characteristics of the partner pool (i.e. mixing) may be more important than the characteristics of the individual partnership[30].

There is a dearth of information regarding specific sexual behaviours and protective behaviours in middle-aged adults. Much of the literature focuses on young adults and adolescents, though a small but growing body of literature is developing on older adults. Data from the National Household Survey on Drug Abuse revealed that, among U.S. men and women aged 35 years or older who had sex in the last year, 12.1% used a condom at last sex[31]. Research on virginity using National Survey of Family Growth data indicated that almost 16% of men and almost 13% of women age 40-45 had never engaged in penile-vaginal intercourse,[32] though many of these would have engaged in other forms of sexual activity. Of single U.S. adults age 40-69, 22% reported having sexual intercourse consistently once a week or more and another 37% reported having had sexual intercourse within the past six months[23]. It is unknown how many engaged in non-intercourse sexual behaviours that could result in herpes transmission. Herpes may be transmitted through a wide variety of behaviours, as viral shedding may occur not only genitally but also orally and on the thighs and buttocks, and transmission commonly occurs during asymptomatic as well as symptomatic periods[33].

A substantial difference between HSV-2 seropositivity and self-reported prevalence was apparent in our analysis, with self-reported genital herpes prevalences remaining low for all groups. Since it is unlikely that this discrepancy is driven by a greater proportion of asymptomatic cases among older, Black and/or unpartnered U.S. residents, it may represent either a greater proportion of undiagnosed cases, a higher reluctance to self-report among members of these groups, or a lack of access to health care for certain groups. It may also represent a lack of recognition of any symptoms as herpetic, as seen in earlier studies[34]. For all groups, only a minority of those with HSV-2 acknowledged genital herpes infection, and most infected individuals were unaware of their status.

Unfortunately, because middle-age and older adults fall outside of an already-identified "high risk group" for STIs - most of which were developed around modes of transmission for HIV - individuals who are actually "at risk" fall through the cracks of targeting strategies for education, diagnosis, and prevention. Once past adolescence and young adulthood, adults are not often considered as being at significant risk for STIs, and physicians do not do as thorough a work up in older patients as they would younger ones[35]. Patients themselves do not often bring up such issues as they are also unlikely to perceive themselves at risk of STIs, even where their behaviour indicates otherwise[36,37]. Moreover, an HIV-centric focus on penile-vaginal intercourse and anal intercourse as risky behaviours due to fluid exchange can obscure assessment of risk for other STIs where risks associated with sexual practices differ from HIV. A broad repertoire of sexual or sensual acts that do not include penile-vaginal or anal intercourse may still produce risk of transmitting HSV-2.

While HSV-2 does not have the broad effects on mortality and morbidity that HIV does, genital herpes can lead to adverse psychological and social effects as well as physical symptoms, and should be taken seriously. Physically, herpes can be mild or can cause repeated painful sores that negatively affect daily life activities. It can also increase the risk of subsequent HIV infection. For childbearing women herpes infection can impact delivery, requiring interventions such as caesarean section to avoid neonatal infection. A diagnosis with genital herpes may also cause psychosocial and psychological distress,[38] including feelings of betrayal, contamination, loss of self-esteem and shame,[39] though the distress experienced may be short-term[40,41]. Interpersonal relationships may be damaged, with the stigma of herpes diagnosis causing difficulty in disclosing status to potential sex partners or in seeking care[41]. A lack of understanding of asymptomatic disease can damage existing relationships as blame for a newly diagnosed infection is based on errant assumptions[39]. Moreover, in a recent meta-analysis of general population cohort studies, it was found that in areas where HSV-2 is highly prevalent, infection was associated with a tripling in the odds of acquiring HIV[42]. Despite these potentially negative consequences, concerns about psychosocial burden should not prevent testing for HSV-2 in clinical practice[43].

This analysis forces us to recognize that a high proportion of adults are seropositive for HSV-2, even a majority in some groups. Given the commonality of this STI, and concern that prevalence may be even higher among some groups of older adults not included in this study, it may be time to increase our ability to discuss infection and decrease stigma around genital herpes. The identification of non-young adults as a group that is at legitimate risk of HSV-2 could enhance understanding that different STIs have differing epidemiologic profiles. Increased diagnostic suspicion regarding lesions could also lead to increased diagnosis of HSV-2, and greater avoidance of sexual contact during symptomatic periods.

## Conclusion

Whereas sexually transmitted infections are often perceived as issues for young adults and specific high-risk groups, the chronic, viral nature of HSV-2 results in accumulation of prevalence with age, especially among those not in married/cohabitating relationships. The disproportionate burden of HSV-2 among Black Americans appear to be due to early incidence by age 20-24, rather than to a more rapid increase across the 20-49 age range of this study. Increased odds of HSV-2 with age did not correspond with increases in self-reported genital herpes, which remained low. Adults who initiate new relationships thus have a substantial probability of either coming into contact with a new partner who is HSV-2 infected, or of having unrecognized HSV-2 infections themselves. Adults and their physicians should be aware of HSV-2 in order to better recognize its symptoms, appropriately treat, and prevent transmission.

## List of abbreviations used

HSV-1: herpes simplex virus type 1: HSV-2: herpes simplex virus type 2: MEC: Mobile Examination Center: NHANES: National Health and Nutrition Examination Survey: STI: sexually transmitted infection.

## Competing interests

The authors declare that they have no competing interests.

## Authors' contributions

GB conceptualized the study, conducted the regression analyses, and drafted much of the manuscript. NK conducted all other analyses, and participated in drafting and editing of the manuscript. TC drafted the background section, participated in analysis decisions, and edited the manuscript. All authors read and approved the final manuscript.

## Pre-publication history

The pre-publication history for this paper can be accessed here:

http://www.biomedcentral.com/1471-2334/10/359/prepub
